# In Situ Growth Method for Large-Area Flexible Perovskite Nanocrystal Films

**DOI:** 10.3390/ma17143550

**Published:** 2024-07-18

**Authors:** Xingting Zhou, Bin Xu, Xue Zhao, Hongyu Lv, Dongyang Qiao, Xing Peng, Feng Shi, Menglu Chen, Qun Hao

**Affiliations:** 1 School of Optics and Photonics, Beijing Institute of Technology, Beijing 100081, China3120225337@bit.edu.cn (H.L.); qhao@bit.edu.cn (Q.H.); 2 Laboratory of Science and Technology on Integrated Logistics Support, National University of Defense Technology, Changsha 410073, China; dyqiao@nudt.edu.cn (D.Q.); shifeng@nudt.edu.cn (F.S.); 3 College of Intelligence Science and Technology, National University of Defense Technology, Changsha 410073, China

**Keywords:** perovskite films, in situ growth, large scale

## Abstract

Metal halide perovskites have shown unique advantages compared with traditional optoelectronic materials. Currently, perovskite films are commonly produced by either multi-step spin coating or vapor deposition techniques. However, both methods face challenges regarding large-scale production. Herein, we propose a straightforward in situ growth method for the fabrication of CsPbBr_3_ nanocrystal films. The films cover an area over 5.5 cm × 5.5 cm, with precise thickness control of a few microns and decent uniformity. Moreover, we demonstrate that the incorporation of magnesium ions into the perovskite enhances crystallization and effectively passivates surface defects, thereby further enhancing luminous efficiency. By integrating this approach with a silicon photodiode detector, we observe an increase in responsivity from 1.68 × 10^−2^ A/W to 3.72 × 10^−2^ A/W at a 365 nm ultraviolet wavelength.

## 1. Introduction

Metal halide perovskites represent a new generation of luminescent materials known for their unique photoelectric traits, which include adjustable band gap, exceptional color purity, and high photoluminescent quantum yield (PLQY). These distinctive features present numerous opportunities for optoelectronic applications such as illumination and displays, which bring about significant implications and challenges for traditional light-emitting technologies and devices [1,2,3,4]. At the same time, halide perovskites are utilized in practical applications across various devices to fabricate high-quality perovskite nanocrystal (PNC) films. Despite recent progress in photoelectric performance, the practical application of PNCs is hindered primarily by challenges relating to quantum efficiency and stability. The luminous characteristics of PNCs are mainly affected by film defects, surface roughness, and the aggregation of nanocrystals [5,6,7].

In recent years, advanced PNC-based light-emitting films or devices have been successfully integrated not only on small area rigid panels but also on large area flexible and/or wearable electronic devices [8,9,10,11]. Most current methods utilize prefabricated nanoparticles fused with polymers to form ligand-free films. In that case, the perovskite is only partially protected, resulting in poor passivation of surface defects and, consequently, low PLQY and stability [12]. To address this issue, magnesium ion doping can be employed to further passivate crystal defects in perovskite and enhance its luminous efficiency [13]. Nevertheless, colloidal synthetic PNCs tend to aggregate and precipitate in solvents, which makes it challenging to produce uniform and high-quality films for optoelectronic devices. Therefore, the in situ growth of PNCs within a polymer matrix represents a promising approach for high-quality PNC films with improved stability. Although various polymer materials and undoped PNCs have been utilized as basic units for constructing hybrid PNC–polymer structures, the straightforward in situ growth of large-area transition metal-doped PNC materials within polymer networks has not been extensively investigated.

Large-area flexible perovskite thin films represent a significant technological advancement that is poised to enable a wide range of innovative electronic applications. Their unique material properties make them exceptionally well suited for use in flexible solar cells, which can harness the high photovoltaic conversion efficiency of perovskites while benefiting from the lightweight, foldable, and conformable form factors enabled by the flexible thin film structure.

This study presents a large-area, in situ growth technique for Mg^2+^-doped PNC films. The process involved incorporating perovskite precursors and Mg^2+^ dopants into a polymethyl methacrylate (PMMA) solution to achieve the in situ growth of Mg^2+^-doped PNCs, as opposed to using pre-prepared nanocrystal suspensions for casting thin films. This method effectively passivated surface defects in the PNC films, improving crystallinity and uniformity while preventing aggregation and precipitation. The resulting film demonstrated stable fluorescence emission that can be adjusted by varying the concentration of Mg^2+^ doping. Integration with a silicon photodiode detector showed an improvement in the responsivity from 1.68 × 10^−2^ A/W to 3.72 × 10^−2^ A/W at a wavelength of 365 nm.

## 2. Materials and Methods

### 2.1. Materials

The following materials used in this study were purchased from Adamas-Beta (Shanghai, China): lead bromide (PbBr_2_, 99%), cesium bromide (CsBr_2_, 99%), N,N-dimethylformamide (DMF, 99.9%) magnesium bromide (MgBr_2_, 98%). Polymethyl methacrylate (PMMA, 99%) was purchased from Aladdin (Shanghai, China). All chemicals were used without further purification.

### 2.2. Methods

#### 2.2.1. Preparation of PMMA Precursors Solution

A total of 1 g of PMMA was combined with 3 mL of DMF in a vial. A magnetic stir bar was added, and the mixture was stirred at 1600 rpm at 100 °C for over 1 h to ensure complete mixing.

#### 2.2.2. Preparation of Stock Solution

A total of 0.30 mmol MgBr_2_ (0.055 g) was dissolved in 3 mL DMF by heating the mixture at 90 °C for 2 h to obtain an opaque solution, while 1.153 mmol CsBr (0.2834 g) and 1.901 mmol PbBr_2_ (0.6973 g) were each dissolved in 6 mL DMF using a magnetic stir bar. Both mixtures were stirred at 1500 rpm at 60 °C for 12 h.

#### 2.2.3. Synthesis of CsPb_1−x_Mg_x_Br_3_@PMMA Films

Before use, the CsBr and PbBr_2_ solutions were preheated for 10 min. Some precipitates formed in the solutions, so the supernatants were utilized. A total of 300 μL of the CsBr solution was added to the PMMA precursors solution along with a magnetic stir bar. The mixture was stirred at 1600 rpm at 100 °C for 12 h. Subsequently, 150 μL of the PbBr_2_ solution and 40/80/120/160 μL of the MgBr_2_ solution were added to the mixture.

**Figure 1:**
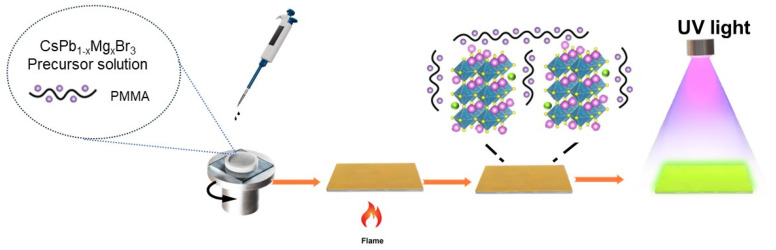
Schematic preparation procedure of Mg^2+^-doped CsPbBr_3_@PMMA film.

Before preparing the film, the glass substrate was underwent 5 min sequential ultrasonic cleaning in acetone. Finally, the substrate was dried using an air gun. As shown in Figure 1, the prepared solution was drawn up using a disposable pipette and uniformly spread onto the glass substrate. Then, the coated substrate was placed on a hot plate set at 90 °C. The sample was exposed to UV light, and the crystal growth process was observed.

### 2.3. Characterization

#### 2.3.1. Characterization via X-ray Diffraction (XRD)

The X-ray diffraction (XRD) diffractometer utilized in this study for polycrystalline film characterization was the PAN analytical instrument (Almelo, The Netherlands). The set-up included a tube voltage set at 40 kV, with a Cu target material selected, that emits radiation at a wavelength of 1.540598 Å. Scanning was conducted within an angular range of 10° to 90° at a rate of 6°/min, with a step size of 0.02°.

#### 2.3.2. Fourier Transform Infrared (FTIR)

The Fourier transform infrared (FTIR) spectra of the samples were recorded using a Ying Sa Optical Instruments FOLI20 (Fluorsa Optics, Shanghai, China). The CNS film was grown in situ on a clean glass sheet and tested.

#### 2.3.3. Fluorescence Spectrum Characterization

The photoluminescence (PL) spectra were measured using the F-380 fluorescence spectrometer (Tianjin Gangdong Sci. & Tech. Development Co., Ltd., Tianjin, China). The ultraviolet (UV)-visible (Vis) absorption spectra of the composite films were detected on a UV-Vis spectrophotometer (N4S) (Shanghai Yi Tian Scientific Instrument Co., Ltd., Shanghai, China). The time-resolved PL (TRPL) decay curves were detected using the FLS1000 fluorescence spectrophotometer (Edinburgh, UK).

#### 2.3.4. Thickness and Surface Roughness of the Film

The thickness of the CsPb_1−X_Mg_x_Br_3_ film was measured using a stylus profiler (Alpha-Step D-300, Shanghai, China). To monitor the evolution of surface quality, we employed a white light interferometer to observe the surface morphology of single-crystal silicon carbide. Specifically, a commercial Zygo New View 700s (Middlefield, CT, USA) white light interferometer with lens magnifications of 10× and 50× was utilized; the data resolution for these magnifications was set at 1.5 μm and 7.5 μm respectively, while the detection range covered an area of 468 μm × 351 μm. The VeriFire Asphere laser wavefront interferometer by Zygo was used to detect low-frequency surface errors.

#### 2.3.5. PNC-Si Photodetector Characterization

The I–V curve of the silicon detector was measured using the source meter Keithley 2602B (Shenzhen, China). The response time of the chip was measured using an oscilloscope (Tektronix, Shanghai, China). Before electrical testing, a layer of CsPbBr_3_ thin film was deposited onto the silicon detector. For the I–V curve measurement, the coated silicon detector was positioned under a 365 nm UV lamp. The I–V curve of the coated detector was then measured and compared to that of an uncoated silicon detector under the same UV illumination. For the response time measurement, the silicon detector was connected to a trans-impedance amplifier circuit. A modulated 405 nm laser was directed towards the detector. The response time of the chip was then measured by the oscilloscope.

## 3. Results

**Figure 2:**
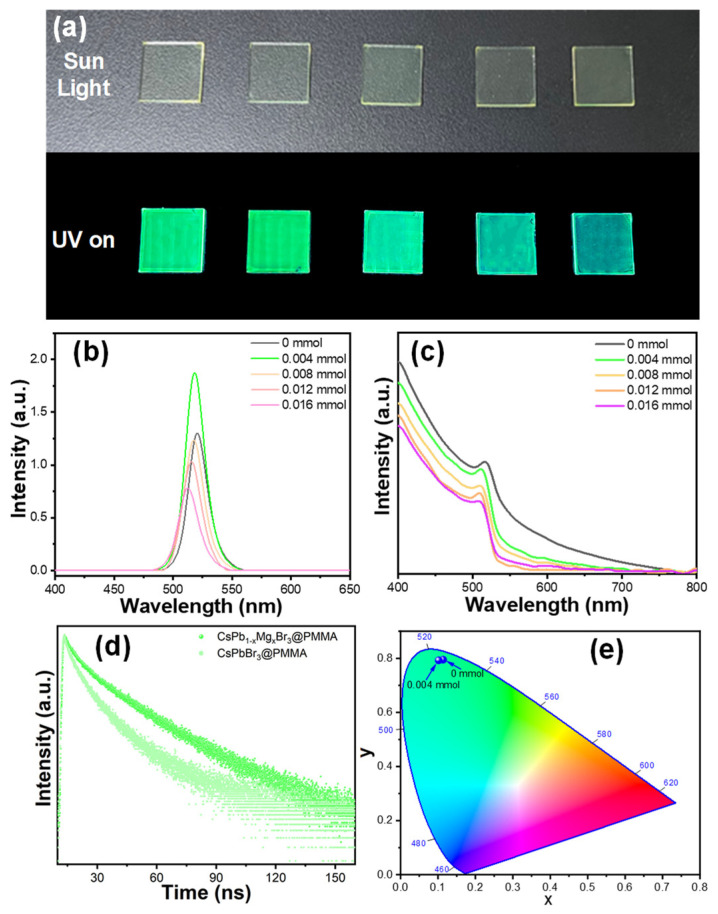
(**a**) A photo of CsPbBr_3_@PMMA with varying concentrations of Mg^2+^ under both daylight and fluorescence conditions; (**b,c**) the PL emission spectra and UV-Vis absorption spectra of CsPb_1−X_Mg_x_Br_3_@PMMA films; (**d**) time-resolved PL decay spectra; (**e**) Chromaticity coordinate diagram of PL emissions of CsPb_1−X_MgxBr_3_@PMMA films.

The optical characterization of CsPbBr_3_@PMMA doped with Mg^2+^ at various concentrations was analyzed through photoluminescence (PL) spectra and UV-visible absorption spectra, as shown in Figure 2. In Figure 2a, the daylight and fluorescence images of CsPbBr_3_ composite films with different Mg^2+^ concentrations under a 365 nm UV lamp are depicted. The sample with 0.004 mmol of Mg^2+^ shows the highest luminescence performance, with luminescence intensity decreasing as Mg^2+^ concentration increases. Under excitation by a 365 nm UV lamp, the emission peak shifts from 520 nm to 511 nm with the addition of 0.016 mmol Mg^2+^ (Figure 2b), while the UV-visible light absorption peak shifts from 520 nm to 502 nm (Figure 2c). This blue shift is believed to be due to the distinct ionic radii of Mg^2+^ and Pb^2+^, causing structural damage in quantum dots at higher Mg^2+^ concentrations [14,15]. This disruption leads to the inability to form a three-dimensional structure, reducing the quantum dot size and resulting in a blue shift in emission peak wavelength. The luminescence intensity increases by approximately 143% compared to samples without magnesium.

The impact of vacancy defects resulting from the loss of surface halides on photogenerated excitons cannot be overlooked entirely. These vacancies create trap states within the band gap, capturing excitons and increasing their non-radiative recombination, which leads to a low PLQY in the sample. Introducing a small amount of doping disrupts the symmetry of perovskite quantum dots, reducing the overlap between electron and hole wave functions and extending the average carrier lifetime. Moreover, studies have shown that substituting smaller cations such as Ni^2+^ and Cu^2+^ for Pb^2+^ can increase the defect formation energy of quantum dots while decreasing their surface defect density, thereby significantly improving their optical properties [16,17,18,19,20].

To investigate the impact of Mg^2+^ doping on CsPbBr_3_@PMMA exciton recombination dynamics, we analyzed the attenuation curve of the time-resolved photoluminescence decay spectrum using a 365 nm light source for excitation and monitored it at 520 nm (Figure 2d and Table 1). The curve was fitted accurately with a double exponential function.

The shorter fitting lifetime is attributed to exciton trapping, while the longer fitting lifetime indicates bounded exciton recombination. A longer lifetime suggests fewer surface trap states in the sample and a higher proportion of radiation transitions. The average lifetime of CsPbBr_3_@PMMA film is 17.57 ns, but with the addition of 0.004 mmol Mg^2+^ during in situ growth, the average lifetime increases by 32.22 ns. This prolonged average lifetime is a result of Mg^2+^ passivating surface defects in CsPbBr_3_, preventing non-radiative electron transitions and enhancing luminescence emission [21]. These results are consistent with changes observed in PL intensity. The addition of Mg^2+^ causes a shift in the corresponding CIE color coordinates, as depicted in Figure 2e, from (0.11, 0.80) blue to (0.09, 0.78). The average lifetime can be calculated by the time-resolved function, where *τ*_1_ and *τ*_2_ represent the decay time of the PL emission, and *A_1_* and *A_2_* are the fractional contributions of the decay components [22].

(1) $$A\left(t\right)=A_{1}e^{\frac{-t}{\tau_{1}}}+A_{2}e^{\frac{-t}{\tau_{2}}},$$

**Figure 3:**
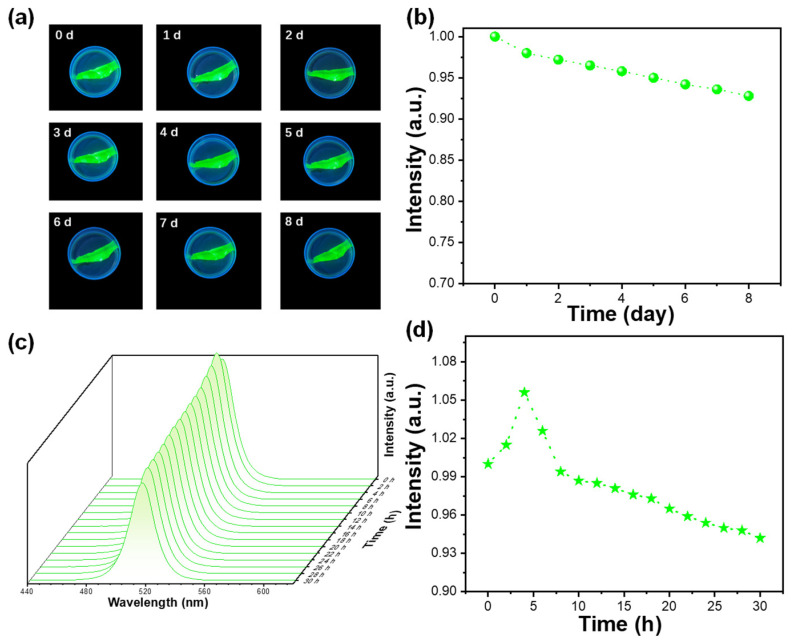
(**a**) Fluorescent photos of water-immersed CsPb_1−X_Mg_x_Br_3_@PMMA film at different time points; (**b**) relationship between immersion time in water and normalized PL; (**c**) light stability characterization; (**d**) the relationship between the irradiation time of UV lamp and the normalized PL.

The water stability and light stability of CsPb_X_Mg_1−X_Br_3_@PMMA were investigated. Exposed CsPbBr_3_ decomposes rapidly in water, leading to a quick reduction in emission intensity. However, green emission from CNS thin films shows long-lasting stability. In Figure 3a, the luminescence intensity of a sample soaked in water for 8 days remains relatively unchanged. Additionally, as shown in Figure 3b, even after 8 days, the luminescence intensity retains 94% of its initial value, demonstrating excellent water stability of the prepared CNS thin films. The photostability of CNS thin films is typically affected by light-induced surface reactions and long-range ion migration within the sample. Exposed CsPbBr_3_ film shows significant fluorescence quenching when continuously exposed to ultraviolet light for up to 30 h. In contrast, as depicted in Figure 3c,d, the CNS thin film exhibits an initial increase followed by a gradual decrease in photoluminescence over time; however, at the 30 h time point, the emission intensity remains above 90% of its initial strength. Short-duration UV light provides enough energy for ions within CNS thin films to undergo short-range migration and effectively passivate defects, thus preventing photoinduced aggregation. Prolonged exposure to ultraviolet light, however, may disrupt long-range migration pathways within quantum dots, leading to increased surface defects and decreased emission intensity in CNS thin films.

**Figure 4:**
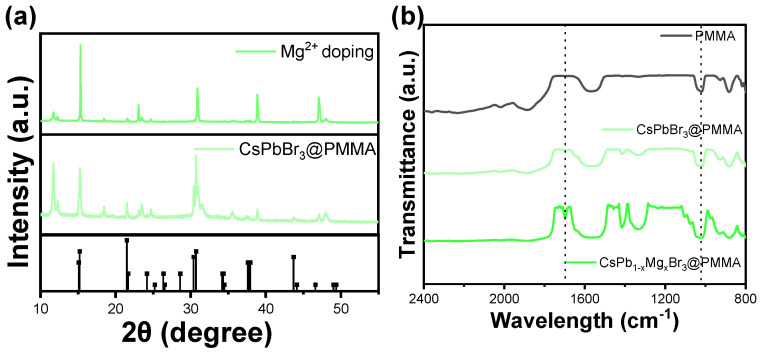
(**a**) XRD spectra of the Mg^2+^−doped CsPbBr_3_@PMMA composite films; (**b**) relationship between immersion time in water and normalized PL.

**Figure 5:**
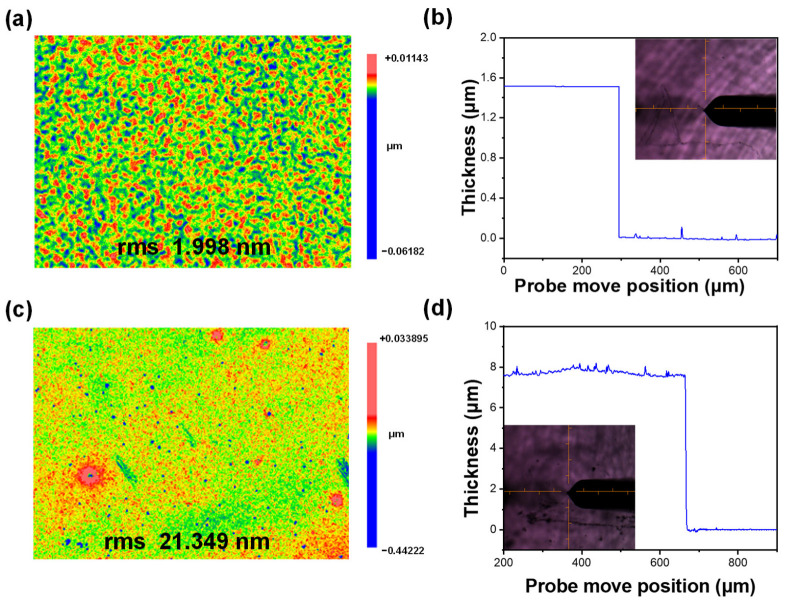
(**a**,**b**) Surface roughness and thickness of sample 1 measured using white light interferometer and stylus profiler; (**c**,**d**) surface roughness and thickness of sample 2 measured using white light interferometer and stylus profiler.

The structure and presence of impurities in the prepared CsPb_1−x_Mg_x_Br_3_ sample were determined through XRD analysis, as illustrated in Figure 4a. Different levels of Mg^2+^ doping are examined to understand their influence on the XRD pattern of CsPb_1−x_Mg_x_Br_3_. The XRD analysis shows clear diffraction peaks at 15.11°, 21.55°, and 30.51°, aligning well with the standard card (JCPDS no. 54-0752) and corresponding to crystal planes (001), (010), and (002) respectively, indicative of a cubic phase in the synthesized perovskite quantum dots [23]. Importantly, the diffraction peak of the Mg^2+^-doped sample shifts noticeably towards higher angles due to crystal contraction resulting from the smaller ionic radius of Mg^2+^ compared to Pb^2+^. Additionally, no new diffraction peaks are detected in the XRD pattern of doped CsPb_1−x_Mg_x_Br_3_ samples, indicating the absence of other impurities. The interaction between PMMA, Mg^2+^, and CsPbBr_3_ perovskite was studied using Fourier transform infrared spectroscopy (FT-IR). In Figure 4b, the absorption peak at 1723 cm^−1^ is attributed to the carbonyl group (C=O) of the PMMA molecule. Interestingly, even after the passivation of PMMA on the perovskite film, the characteristic vibration of C=O can still be observed with a redshifted peak at 1713 cm^−1^ [24,25,26]. This indicates the formation of coordination bonds between the carbonyl group in PMMA and Pb^2+^. Furthermore, a new infrared peak is seen at 721 cm^−1^ in the Mg^2+^-modified CQD film, which is attributed to its characteristic vibrations, confirming the presence of Mg^2+^ in the CsPbBr_3_@PMMA film [27]. The surface roughness and thickness of CNS thin films are crucial factors affecting their luminescence properties. In this study, we further investigated how the spin coating process influenced the in situ growth of CNS films in terms of surface roughness and thickness. By using a homogenizer at 3000 rpm, we successfully fabricated the CNS thin film for sample 1, as shown in Figure 5a.

Subsequently, we quantify the surface roughness using PV (peak valley) and RMS (root mean square) parameters, which reveal a surface roughness of 1.998 nm RMS [25]. Furthermore, a stylus profiler test indicates a film thickness of 1.5 microns (Figure 5b). On the other hand, utilizing a homogenizer at 1500 rpm results in the CNS film for sample 2, showing an identical RMS surface roughness of 0.25 nm but with an increased thickness of 7.8 microns. These findings indicate that increased film thickness corresponds to a rise in surface roughness, which ultimately impacts surface quality.

**Figure 6:**
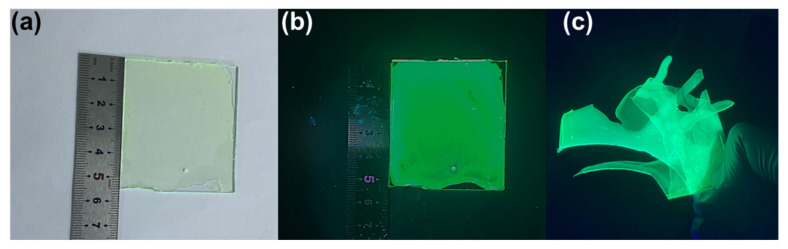
(**a**,**b**) Photographs of CsPb_1-x_Mg_x_Br_3_@PMMA film growing on quartz glass substrates under daylight and ultraviolet irradiation, respectively; (**c**) photographs of the CsPb_1-x_Mg_x_Br_3_@PMMA film at bending degree.

The preparation of large and uniform CNS thin films using traditional spin coating techniques presents a significant challenge. In this research study, we employed a spray in situ growth method to fabricate CsPb_1−x_Mg_x_Br_3_@PMMA films. The precursor solution was evenly distributed on a quartz glass substrate, followed by annealing at 90 °C for 90 s to form a CsPbBr_3_ nanocrystalline film. Notably, the formation of CsPbBr_3_ nanocrystals occurred during the annealing process, eliminating the laborious steps involved in preparing and purifying perovskite materials. Compared to other PNC thin films, as shown in Figure 6a,b and Table 2, our spray technology enables the in situ growth of CsPb_1-x_Mg_x_Br_3_@PMMA films with dimensions exceeding 5.5 cm × 5.5 cm on various substrates (glass, sapphire, and silicon), while maintaining good transparency. When exposed to UV light at 365 nm, these films exhibit a vivid green fluorescence (Figure 6c). Furthermore, the CsPbBr_3_@PMMA films display excellent flexibility. The CsPb_1−x_Mg_x_Br_3_@PMMA films demonstrate exceptional uniformity due to the strengthened chemical interaction between PMMA, Mg^2+^, and PbBr_2_. This interaction effectively inhibits the rapid aggregation of perovskite clusters and improves the consistency of perovskite films. These findings support the promising potential of CsPb_1-x_Mg_x_Br_3_@PMMA films.

**Figure 7:**
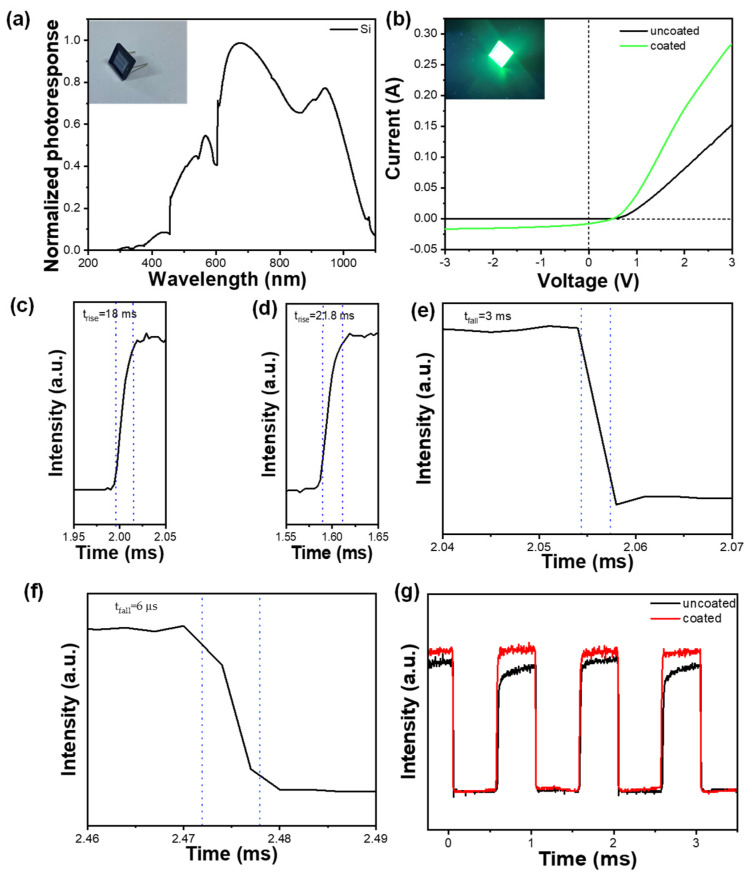
(**a**) Response spectrum of pure silicon detector; (**b**) I−V curve of silicon detector with or without coating (black for no coating, green for coating); (**c**) response time when the detector has a film; (**d**) response time when the detector has no membrane; (**e**) decay time when the detector has a film; (**f**) decay time when the detector has no membrane (The blue dashed line represents the time at which 90% or 10% of the steady-state value is located); (**g**) transient response curve under ultraviolet light with wavelength of 405 nm.

To explore the photoelectric properties of CNS thin films, a layer of the film was grown in situ on the surface of a silicon detector. The noise level at V_r_ = 0 V is about 0.1 nA, which is negligible. The response spectrum of the pure silicon detector obviously indicates its low sensitivity to UV wavelengths (Figure 7a), particularly around 400 nm and below. In Figure 7b, the I–V curve of the silicon detector is displayed under irradiation from a 365 nm UV lamp without coating (black line) and after coating with the perovskite film (green line). The photocurrent of the silicon detector with the perovskite film is significantly enhanced compared to the uncoated detector. The power of the UV light that shines on the detector is about 7.60 W. Utilizing the response formula [32], the responsivity of the silicon detector increases from 1.68 × 10^−2^ A/W to 3.72 × 10^−2^ A/W.

(2) $$R=\frac{I}{P_{I_{t}}},$$

The findings in Figure 7g demonstrate that the perovskite film coating significantly improved the device’s responsiveness when exposed to near-ultraviolet (405 nm) irradiation at a modulation frequency of 1 kHz. Additionally, the coating played a role in shaping the response curve, leading to a reduction in fluctuations in the response peaks. The signal arose and fell over the course of time, as shown in Figure 7c–f, which refer to the time taken for the signal to increase from 10% to 90% or to decrease from 90% to 10% of the steady-state value. The results reveal that the coated surface exhibited a response time of approximately 18 μs and a decay time of approximately 3 μs, whereas the uncoated surface had a response time of about 21.8 μs and a decay time of about 6 μs. The minimal impact on response time suggests that further optimization of the coating process or material composition may be needed to achieve more significant enhancements in this parameter.

## 4. Conclusions

Large-area CsPbBr_3_ perovskite films with controlled thicknesses were successfully synthesized using the in situ growth method. The luminescence intensity of the perovskite thin films was effectively regulated by Mg^2+^ doping, resulting in a maximum increase of 143%. The optimization mechanism of Mg^2+^ doping was determined via XRD and FTIR analysis, showing that Mg^2+^ addition passivates surface defects in the perovskite film. Furthermore, the PL intensity of CsPbBr_3_ perovskite remained stable for an extended period in aqueous environments and exhibited high photostability under ultraviolet lamp irradiation. The CsPbBr_3_ perovskite film displayed strong UV absorption and high visible light transmittance, enabling indirect detection of the ultraviolet region when coupled with a silicon-based detector. The device’s rise time was measured to be 21.8 μs, indicating a superfast response speed. Specifically, at a UV wavelength of 365 nm, the responsivity of the silicon photodiode detector increased from 1.68 × 10^−2^ A/W to 3.72 × 10^−2^ A/W. This study presents a simple method for achieving large-area CsPbBr_3_ films with smooth surfaces and highlights the potential of utilizing a CsPbBr_3_ silicon heterojunction as a promising alternative for fast-response light detection.

## Figures and Tables

**Table 1:** The fitted time-related PL decay results of CsPbBr_3_@PMMA and CsPb_1−X_Mg_x_Br_3_@PMMA films.

Sample	*A*_1_	*τ*_1_ (ns)	*A*_2_	*τ*_2_ (ns)	*τ*_avg_ (ns)	x^2^
CsPbBr_3_@PMMA	0.30	5.24	0.76	18.92	17.57	0.99
CsPb_1−X_Mg_x_Br_3_@PMMA	0.24	5.16	0.83	33.54	32.22	0.99

**Table 2:** Size comparison of large perovskite films.

Name	Size (cm^2^)	Ref.
(FAPbI_3_)_0.95_(MAPbBr_3_)_0.05_PSCs	25 cm^2^	[28]
(Cs_0.05_FA_0.81_MA_0.14_)Pb(I_0.86_Br_0.14_)_3_ (CsFAMA)	1.02 cm^2^	[29]
MAPbI_3_	4 cm^2^	[30]
MAPbBr_3_PNCs@polymer	17 cm^2^	[31]
CsPb_1−X_Mg_x_Br_3_@PMMA	30.25 cm^2^	This work

## Data Availability

The original contributions presented in the study are included in the article, further inquiries can be directed to the corresponding author.
